# Evaluation of Antimicrobial Effects of Different Concentrations of Triple Antibiotic Paste on Mature Biofilm of *Enterococcus faecalis*

**DOI:** 10.15171/joddd.2015.027

**Published:** 2015-09-16

**Authors:** Mohammad Frough Reyhani, Saeed Rahimi, Zahra Fathi, Sahar Shakouie, Amin Salem Milani, Mohammad Hossein Soroush Barhaghi, Javad Shokri

**Affiliations:** ^1^Dental and Periodontal Research Center, Tabriz University of Medical Sciences, Tabriz, Iran; ^2^Associate Professor, Department of Endodontic, Faculty of Dentistry, Tabriz University of Medical Sciences, Tabriz, Iran; ^3^Professor, Department of Endodontic, Faculty of Dentistry, Tabriz University of Medical Sciences, Tabriz, Iran; ^4^Postgraduate Student, Department of Endodontic, Faculty of Dentistry, Tabriz University of Medical Sciences, Tabriz, Iran; ^5^Assistant Professor, Department of Endodontic, Faculty of Dentistry, Tabriz University of Medical Sciences, Tabriz, Iran; ^6^Assistant Professor, Department of Microbiology, Faculty of Medicine, Tabriz University of Medical Sciences, Tabriz, Iran; ^7^Associate Professor, Department of Pharmaceutics, Faculty of Pharmacy, Tabriz University of Medical Sciences, Tabriz, Iran

**Keywords:** Anti-bacterial agents, Enterococcus faecalis, root canal medicaments, triple antibiotic paste

## Abstract

***Background and aims.*** Triple antibiotic paste (TAP) is widely used in endodontics for root canal disinfection, particularly in regenerative procedures. The aim of this in vitro study was to evaluate the antimicrobial effects of different concentrations of TAP at 1-, 2-, 3-, and 4-week intervals on mature *Enterococcus faecalis * biofilm.

***Materials and methods.*** A total of 287 extracted one-rooted human central incisors were infected with E. faecalis ATCC 29212 after removing the crown and preparation. The root canal space was filled with one of the 0.01-, 0.1-, 1-, 10-, 100-, and 1000-mg/mL concentrations of TAP or normal saline (control). The root canal dentin was sampled after 1, 2, 3, and 4 weeks. The dentinal shavings were cultured on Mueller-Hinton agar plates after serial dilutions. The classic colony-forming unit (CFU) counting technique was used to determine remaining bacterial counts. Data were analyzed by using the two-way ANOVA, post hoc Tukey tests and one-way ANOVA (P<0.05).

***Results.*** TAP completely eliminated *E. faecalis * biofilms at all the intervals at concentrations of 1000, 100, and 10 mg/mL, whereas 1-, 0.1-, and 0.01-mg/mL TAP resulted in significant reduction of CFU means compared with the control group. There were no statistically significant differences between the four time intervals.

***Conclusion.*** Use of lower concentrations of TAP at short term could eradicate *E. faecalis* biofilm and decrease high-concentration side effects.

## Introduction

*Enterococcus faecalis* is the chief colonizing microorganism in endodontic infections and the most important bacterial species in refractory infections of the root canal system.^1^ A unique characteristic of this bacterial species is its ability to form biofilms; *E. faecalis* biofilms are resistant against antibodies, antimicrobial agents and phagocytes.^2^

Treatment of infections in the root canal space of immature teeth is a challenge in endodontics. Since root canal walls are weak in such teeth, mechanical instrumentation should be limited and emphasis should be placed on irrigation solutions and intracanal medicaments.^3^ Traditional treatment modalities, including apexification with calcium hydroxide and apical barrier technique with MTA do not allow the root to continue to grow, making the root structure prone to fracture.^4-6^Therefore, there is a change in the treatment protocols of such teeth toward regenerative processes. Endodontic regenerative processes are feasible with the use of tissue engineering principles which require spatial orientation of stem cells, signaling molecules and scaffolds as prerequisites.^7^ It has been reported that the remnants of Hertwig epithelial sheath or the remnants of Malassez rest cells are resistant to periapical infections; therefore, signaling networks from these residual cells in immature and non-infected non-vital teeth stimulate different stem cells, including apical papilla stem cells, bone marrow and pluripotent stem cells of the pulp to form dentin and help the normal root maturation.^7^ Prerequisites for regenerative treatments include the presence of a sterilized environment, a matrix for cellular growth and a hermetic coronal seal to prevent re-contamination.^8,9^ In this context, the most commonly used and studied agent for disinfection of the root canal is triple antibiotic paste (TAP), which is a mixture of metronidazole, ciprofloxacin and minocycline. It has been demonstrated that this material is effective against the majority of endodontic pathogens.^10^ However, the concentration of this material used in various studies is mainly empirical and in most cases a thick paste (1000 mg/mL) has been used. Some studies have shown that the topical use of TAP at a high concentration might have a toxic effect on the host, resulting in cellular death, especially the stem cells, which disrupts tissue regeneration processes.^11,12^ Another important consideration is the time needed to apply TAP within the root canal, which has been reported to range from a few days to several months in different studies.^6,8,13,14^ However, it is not clear at what interval the suggested concentrations can eliminate the intracanal biofilm.

Recent in vitro studies on the effect of antibacterial agents used in root canal treatment against *E. faecalis* have predominantly focused on biofilms because planktonic bacterial culture models cannot completely simulate the in vivo growth conditions in infected root canal systems.^15^The aim of this in vitro study was to evaluate the antibacterial effects of different concentrations of TAP at 1-, 2-, 3-, and 4-week intervals on mature *E. faecalis* biofilms.

## Materials and Methods

### Selection and Preparation of Samples 

A total of 287 extracted one-rooted human central incisors with closed apices were selected. The teeth were stored in 3% chloramine T solution until used for the purpose of the study. All the calculi and periodontal tissue remnants were removed with an ultrasonic device. A disk (D&Z, Diamant, Germany) was used to remove tooth crowns at the coronal third to achieve a root length of 12 mm. Then a #15 K-file (Dentsply Maillefer, Ballagues, Switzerland) was used to determine the working length. In the next stage, #4, #3, #2 and #1 Gates-Glidden drills (Maillefer, Dentsply, Switzerland) were used to prepare root canal walls using the crown-down technique. Then the canals were instrumented up to #60 K-file. Physiologic serum was used for canal irrigation during root canal preparation. The smear layer was removed using 5.25% sodium hypochlorite solution for 3 minutes and 17% EDTA (Pulpdent Corp., MA, USA) for 3 minutes. The final irrigation was carried out with PBS (phosphate-buffered saline solution). Two samples were selected randomly and evaluated under SEM (VEGAII TESCAN, Cranberry, PA) to assess dentinal tubules for formation of *E. faecalis* biofilms.

### Formation of Biofilm

After autoclaving the samples at 121°C and under a pressure of 15 psi for 20 minutes, the samples were incubated in BHI (brain-heart infusion broth) (Merck, Darmstadt, Germany) at 37°C for 24 hours to confirm their sterility. Then each sample was placed within a sterile microtube containing 2 mL of standard suspension of *E. faecalis* (ATCC 29212). To prepare the standard suspension, first the pure bacterial culture was incubated at 37°C in the presence of 10% CO_2_ for 24 hours to isolate the bacteria. Then the new bacteria were cultured in BHI and the bacteial density was adjusted at 10^8^ cells/mL using a spectrophotometer (UV-VISIBLI, Shimadzu, Japan) at an optical density (OD) of 1 at 600 nm according to 0.5 MacFarland standard. In order to make sure of the stability of the culture media and elimination of additional bacterial cells, the broth culture media were changed every other day. The samples were incubated at 37°C during all these steps.

**Figure 1.**
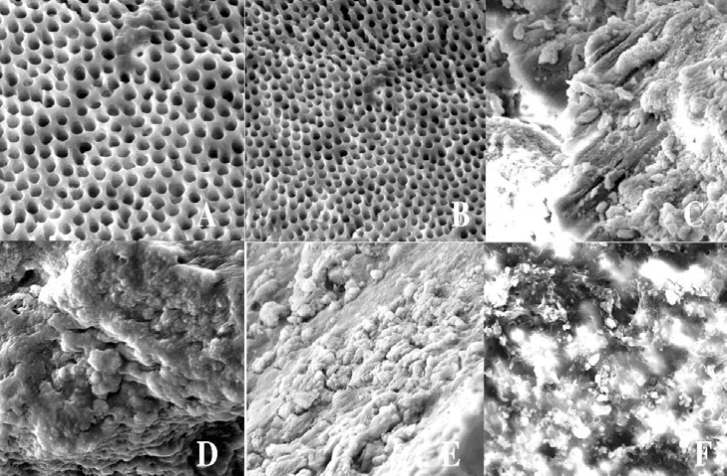
Scanning electron microscopic (SEM) images of root canal dentinal tubules. Appearance of open dentinal tubules after removal smear layer and before E.faecalis incubation ×2000 (A) and ×3000 (B). E. faecalis biofilm to-pography after 6 weeks incubation ×2000 (C and D) and ×3000 (E and F).

After 6 weeks, 5 samples were randomly selected and formation of biofilms was evaluated and confirmed under SEM after preparation of longitudinal cross-sections Figure 1.

### Preparation of Medications 

To prepare TAP, at first equal amounts (weight) of metronidazole, minoycline and ciprofiloxacin were mixed with a mixed base of glycerine, mannitol and colloidal silicone dioxide and diluted serially to achieve the desired concentrations.

### Classification of Samples and Use of Antimicrobial Agents 

After preparation of the samples and production of biofilms, the samples were randomly divided into 7 groups of 40 based on the protocol used:

Group 1: 1000 mg/mL of TAP

Group 2: 100 mg/mL of TAP

Group 3: 10 mg/mL of TAP

Group 4: 1 mg/mL of TAP

Group 5: 0.1 mg/mL of TAP

Group 6: 0.01 mg/mL of TAP

Group 7: normal saline

After placing the medications in the root canals with a Lentulo spiral (Caulk, Milford, DE), the canal orifices were dressed with Cavit (ESPE, Noristown, PA) and each sample was wrapped in a piece of sterile gauze impregnated with PBS and then placed in a sterile microtube which was incubated at 37°C. Each group was randomly divided into 4 subgroups so that each subgroup consisted of 10 samples. Ten samples from each group were evaluated after 1, 2, 3 and 4 weeks, respectively. After the time intervals mentioned above, the samples were irrigated with normal saline solution and placed in a freezer at -25°C for 1 hour for precooling to prevent destruction of *E. faecalis* bacteria due to the heat produced by drilling during the subsequent sampling procedures.

After 24 hours, #6 and #5 Gates-Glidden drills were used to collect 10 mg of dentin chips produced due to scraping of root canal walls. To this end, the drills were placed in the root canal up to 1 mm short of the working length. The dentin chips from each root canal were placed on plates which were weighed by a sensitive electronic weighing machine (ASD Co., LTD, Japan) before and after placing the dentin chips. Then the chips were transferred into test tubes containing 2 mL of sterile physiologic serum and mixed for 20 seconds using whirling movements. Finally, serial dilutions from 10 folds to 10^-7^ were prepared. In the next stage, 100 mL of each dilution were added to 30 plates of Mueller-Hinton cultures and incubated at 37°C for 48 hours. A classic colony-forming unit (CFU) counting technique was used to count *E. faecalis* colonies retrieved.

### Statistical Analysis

CFU counts were converted to Log_10_ and SPSS 15 was used for statistical analysis of data. Two-way ANOVA was used to evaluate differences between the materials and the time intervals used. Post hoc Tukey tests were used for two-by-two comparisons of the groups. One-way ANOVA was used to compare CFU means between the four time intervals. Statistical significance was defined at P<0.05.

## Results

The results of statistical analysis showed that TAP at concentrations of 1000, 100, and 10 mg/mL completely eliminated mature *E. faecalis* biofilms at 1-, 2-, 3- and 4-week intervals.

**Figure 2.**
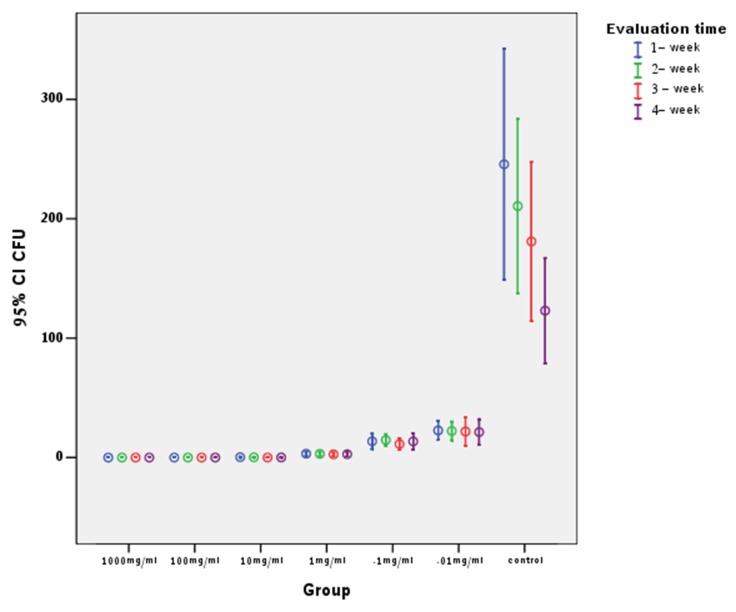
Effect of antibacterial approach against *Enterococcus faecalis*.

Table 1 presents means and standard deviations of CFUs in study groups. Post hoc Tukey test revealed the highest CFU means in group 7, with significant differences from the other 6 groups, whereas other pairwise comparisons indicated no statistically significant differences from each other (Figure 2). Evaluation of the effect of time intervals on CFU means exhibited no statistically significant differences between the four time intervals.

**Table 1:** Mean(±SD) of Colony –forming units of *E. faecalis* in the study groups

**Group**	**1-week**	**2-week**	**3-week**	**4-week**
**1**	0	0	0	0
**2**	0	0	0	0
**3**	0	0	0	0
**4**	3.20(±2.53)	3.10(±2.13)	2.70(±2.06)	2.80(±2.53)
**5**	13.60(±9.16)	14.70(±6.89)	11.30(±6.86)	13.50(±9.72)
**6**	22.70(±11.31)	22.30(±10.92)	21.80(±16.36)	21.30(±15.18)
**7**	245.60(±135.30)	210.6(±101.9)	181(±93.20)	123(±61.70)

## Discussion

Treatment of immature necrotic teeth is a challenge in endodontics and requires special disinfection procedures to achieve regenerative aims.

The results of the present study showed that all the 6 concentrations of TAP used in this study could significantly reduce bacterial counts in comparison with normal saline; however, concentrations of 1000, 100 and 10 mg/mL could completely eradicate mature* E. faecalis* biofilm. In addition, the effect of using this intracanal medicament for 1 week was similar to that of 4 weeks. *E. faecalis* was used in the present study because it has a major role in resistant infections of the root canal system. The biofilm model was used instead of the planktonic model to mimic the clinical scenario and better simulate the in vivo situation.

Since infection of the root canal system has a polymicrobial nature, consisting of aerobic and anaerobic bacteria, use of a combination of several medications yields better results and decreases the potential of spread of resistant bacterial species. A combination which has been used in different studies and has exhibited a high success rate is a mixture of three antibiotics, including metronidazole, ciprofloxacin and minocycline.^16^ Previous studies have shown the antimicrobial activity of TAP against oral bacteria, and its ability to sterilize infected dentin has been exhibited; the present study confirmed the results of previous studies.^17-22^

A combination base composed of glycerine, mannitol and coloidal silicone dioxide was used to prepare different concentrations of TAP. This base was used because it is penetrable to moisture and bacteria can grow in it. This way, there was no interference between the effects of the base and the antibiotics used, and the inhibitory effects were attributed only to the effect of the antibiotics used. Use of mannitol and glycerine resulted in the formation of a paste form and coloidal silicone dioxide was used to decrease adhesion of the paste. Preparation of such a formula facilitated the use of the material within the root canal.

In the present study, concentration multiples of 10 were used because it is easier and more accurate to prepare serial concentrations from the initial concentration; in addition, such concentrations have been evaluated in a study by Ruparel et al^11^in relation to their cytotoxicity, which could be used as a criterion to determine the concentration that could form a balance between antibacterial and cytotoxic effects.

It appears it is important to use a concentration of TAP, which can disinfect the root canal completely and remove the bacterial biofilm on one hand and does not damage the stem cells in the area on the other hand because in the treatment of immature necrotic teeth it is necessary to achieve regeneration and continue maturation of the root.

Hoshino et al^22^reported that 25 µg/mL of this material can eliminate all the isolated bacteria. Also a study by Chuensombat et al^12^ showed that 25 µg/mL of TAP can completely eliminate the bacteria cultured on agar; this difference was probably due to our method in using mature biofilm.

An in vitro study by Ruparel et al^11^, in which 0.01, 0.1, 1,10 and 100 mg/mL concentrations of TAP were used on SCAP cells, showed that with the use of TAP at 10-100 mg/mL less than 20% and with the use of 1 mg/mL cocentration 33-56% of stem cells remained viable and only at concentrations of 0.1-0.01 mg/mL no negative effect was observed on the viability of stem cells of the apical papilla. Therefore, a balance between antibacterial effects and cytotoxicity should be considered.

On the other hand, in addition to minocyclin,s positive effects on modulating host responces by inhibiting collagenases and matrix metaloproteinases, impeding osteoclastogenesis, regulating angiogenesis and substantivity, it binds calcium ions through chelation and produces an unfavorable compound which discolors teeth.^20,23,24^ It has been reported that even a short-term application of minocycline for 24-48 hours, too, results in tooth discoloration.^25^ In the present study, too, severe tooth discoloration was observed at high concentrations of TAP, which was not observed at low concentrations. Therefore, it appears that with lower concentrations of TAP there are no concerns about tooth discoloration due to minocycline. The time necessary for TAP dressing in root canal has been reported to be several days to several months; it has also been reported that the treatment procedure can continue after removal of TAP from the root canal in the absence of symptoms and signs of periradicular conditions.^26^ However, no study has determined at what time interval the paste can completely eliminate the bacteria from the root canals. Based on a study by Chuensombat et al^12^ the cytotoxic effects of TAP are time- and dose-dependent and the effects increase with an increase in exposure duration; therefore, it seems that, time, too, is an important factor in the use of this medication. The results of the present study showed that the effect of 1-week use of this paste is not different from those of 2-, 3- and 4-week. However, more in vivo studies are necessary to validate these findings.

The results of the present study suggest that use of lower concentrations of TAP might decrease tooth discoloration potential and at the same time increase the potential of regeneration due to a decrease in the negative effects on the stem cells in the area; it is possible that 1-week use of this medication might be able to eradicate bacterial biofilms from the root canal space.
